# LncRNA HULC induces the progression of osteosarcoma by regulating the miR-372-3p/HMGB1 signalling axis

**DOI:** 10.1186/s10020-020-00155-5

**Published:** 2020-03-18

**Authors:** Yong Li, Jing-Jing Liu, Jia-Hui Zhou, Rui Chen, Chao-Qun Cen

**Affiliations:** 1 Department of Emergency Medicine and Intensive Care Unit, The Third Xiangya Hospital of Central South University, No. 138 Tongzipo Road, Yuelu District, Changsha, 410013 Hunan Province People’s Republic of China; 2 Department of Intensive Medicine, The Third Xiangya Hospital of Central South University, Changsha, 410013 Hunan Province People’s Republic of China; 3 Department of Orthopedics, The Third Xiangya Hospital of Central South University, Changsha, 410013 Hunan Province People’s Republic of China; 4 Department of Orthopedics, The First Naval Hospital Southern Theater Command, Zhanjiang, 524000 Guangdong Province, People’s Republic of China

**Keywords:** ceRNA, HMGB1, HULC, miR-372-3p, Osteosarcoma

## Abstract

**Background:**

Osteosarcoma is a malignancy that normally affects children, adolescents, and young adults. Although accumulating evidence has demonstrated the importance of HULC in osteosarcoma, little is reported about its functional roles and molecular mechanisms.

**Methods:**

The expression of HULC and miR-372-3p in osteosarcoma tissues was quantified by qRT-PCR. The regulatory roles of HULC and miR-372-3p on cell proliferation, apoptosis, migration and invasion were determined by CCK-8, colony formation, flow cytometry, wound healing, and transwell assays, respectively. The bioinformatics prediction software RAID v2.0 was used to predict the putative binding sites. The interactions among HULC, miR-372-3p and HMGB1 were explored by luciferase assay and western blot assay.

**Results:**

Our results revealed elevated HULC and decreased miR-372-3p expression in both osteosarcoma tissues and cell lines. Overexpression of HULC or knockdown of miR-372-3p promoted osteosarcoma cell proliferation, migration and invasion and induced cell apoptosis. Bioinformatics and luciferase assays verified that HULC directly interacted with miR-372-3p to attenuate miR-372-3p binding to the HMGB1 3′-UTR. Furthermore, mechanistic investigations confirmed that activation of the miR-372-3p/HMGB1 regulatory loop by knockdown of miR-372-3p or overexpression of HMGB1 reversed the in vitro roles of HULC in promoting osteosarcoma cell proliferation, migration and invasion.

**Conclusion:**

Our study is the first to demonstrate that HULC may act as a ceRNA to modulate HMGB1 expression by competitively sponging miR-372-3p, leading to the regulation of osteosarcoma progression, which provides new insight into osteosarcoma diagnosis and treatment.

## Background

Osteosarcoma is a highly aggressive malignancy that normally affects children, adolescents, and young adults. Patients suffering from osteosarcoma may have an onset of pain and swelling in the affected bone. Occasionally, severe pain or other pathologic fracture-associated symptoms occur.(Isakoff et al. 2015; Bielack et al. 2002) Approximately 15 to 20% of patients have clinically detectable metastases at the time of diagnosis.(Isakoff et al. 2015) Unfortunately, the 5-year survival rates are as low as 19%.(Wang et al. 2017a; Harrison et al. 2018) The current treatment of osteosarcoma involves chemotherapy followed by surgical resection, which has largely remained unchanged for more than 30 years, has unsatisfactory effectiveness and provides limited improvement of survival outcomes.(Wang et al. 2017a) This is primarily because osteosarcoma pathogenesis has long been known to be complicated, with few common features between tumours. Therefore, it is urgently necessary to explore novel and clinically relevant biomarkers or targets for osteosarcoma therapeutic use.

Long noncoding RNAs (lncRNAs), defined as genome transcripts having more than 200 nucleotides but not translated into proteins, are associated with various biological processes, such as tumour proliferation and metastasis. Highly upregulated in liver cancer (HULC) is a lncRNA that was first found to be strongly overexpressed in hepatocellular carcinoma. Recently, accumulating evidence has demonstrated the crucial roles of HULC in osteosarcoma. HULC is significantly highly expressed in osteosarcoma compared with normal controls. Its increased expression may act as an independent predictor of poorer survival in osteosarcoma patients. Moreover, the knockdown of HULC inhibits proliferation, migration, and invasion and promotes apoptosis in osteosarcoma.(Wang et al. 2017a; Li et al. 2017; Kong and Wang 2018) All the data suggest that HULC has the potential to be a diagnostic marker or therapeutic target for osteosarcoma.

Gene expression in tumours can be positively or negatively modulated by lncRNAs either through epigenetic transcriptional regulation or posttranscriptional regulation. For the epigenetic mechanism, lncRNAs may interact with the transcription preinitiation complex at the promoter region or directly be base paired with RNA and DNA. For the posttranscriptional mechanism, lncRNAs act as precursors of microRNAs (miRNAs) and compete with endogenous RNAs (ceRNAs) to control cell fate.(Li et al. 2017) Among these regulatory mechanisms, the ceRNA mechanism, which regulates gene expression via miRNA sequestration, has been widely studied and recognized.(Salmena et al. 2011) It has been reported that affecting HER2 levels via miR-331-3p interplay might be the mechanism underlying the oncogenic roles of Hox transcript antisense intergenic RNA (HOTAIR) in gastric cancer.(Liu et al. 2014; Lee et al. 2013) Similarly, another paper demonstrated that the oncogenic functions of lncRNA H19 are attributed to its ceRNA activity to sequester miR-138 and miR-200a in colorectal cancer (CRC).(Liang et al. 2015)

miRNAs are identified as a class of short (18–25 nucleotides in length) noncoding endogenous RNAs. They are involved in gene regulation at the posttranscriptional level by binding to the 3′ untranslated region (3’UTR) of the target mRNAs, leading to target mRNA degradation or transcription inhibition.(Bartel 2004) miRNAs have been demonstrated to play key roles in the cell cycle, proliferation, apoptosis, developmental timing, and metabolism.(Croce and Calin 2005; Bartel 2005; Ardekani and Naeini 2010) Previous studies have revealed that miR-372-3p is associated with dysregulated expression in various tumours. It has been demonstrated that miR-372-3p regulates cell growth and metastasis by targeting FGF9 in lung squamous cell carcinoma.(Wang et al. 2017b) Another report revealed that miR-372-3p inhibited the growth and metastasis of osteosarcoma cells by targeting FXYD6.(Xu et al. 2018) HULC could act as an endogenous sponge antagomir to downregulate miR-372 expression in hepatic cell carcinoma.(Wang et al. 2010) However, whether HULC acts as a ceRNA to reduce miR-372-3p expression in OS needs further exploration.

In this study, we demonstrated a significant upregulation of HULC, which acted as an oncogene, in OS tissue samples and cell lines. After discovering that HULC knockdown can significantly inhibit cell proliferation, invasion, and migration, as well as induce apoptosis, we identified miR-372-3p as an inhibitory target of HULC. By using a luciferase assay as well as qRT-PCR, we determined that HULC knockdown could increase miR-372-3p expression. Furthermore, we observed that miR-372-3p overexpression had similar effects to the low expression of APTR. We subsequently investigated the targets of miR-372-3p and surprisingly identified high mobility group box 1 protein (HMGB1), which was shown to be a direct target of miR-372-3p. HULC knockdown suppressed cell proliferation, invasion, and migration by inhibiting miR-372-3p expression and elevating HMGB1 expression. Thus, we hypothesized that HULC might promote the development of OS by modulating the miR-372-3p/HMGB1 axis, which provides a new potential therapeutic target for osteosarcoma.

## Methods

### Human tissue specimens

A total of 32 pairs of tumour and adjacent normal tissue samples were obtained from osteosarcoma patients who underwent surgery at The Third Xiangya Hospital. The collection of clinical specimens was approved by the Ethics Committee of Central South University and was performed according to the Declaration of Helsinki. Informed consent was obtained from all participants.

### Cell culture

Osteosarcoma cell lines (SAOS-2, U2OS, HOS, MG63) and the human osteoblast cell line hFOB were obtained from American Type Culture Collection (ATCC; Manassas, VA). Dulbecco’s modified Eagle’s medium (DMEM; Invitrogen, USA) containing 10% foetal bovine serum (FBS), 100 U/ml penicillin, and 100 μg/ml streptomycin (Invitrogen, USA) was used to culture all cells at 37 °C and 5% CO_2_.

### Cell transfection and plasmid construction

Mimics NC, miR-372-3p mimics, inhibitor NC and miR-372-3p inhibitor were acquired from RiboBio (Guangzhou, China). The HULC overexpression plasmid pcDNA-HULC and the HULC knockdown shRNA plasmid (sh-HULC) with a corresponding negative control shRNA (sh-NC) were synthesized by GenePharma (Shanghai, China). Lipofectamine 2000 (Invitrogen, USA) was used for the transfection experiments according to the manufacturer’s instructions. Cells were collected for further use after 48 h of transfection.

### CCK-8 assay

The Cell Counting Kit-8 assay (CCK-8; Dojindo, Japan) was used to evaluate cell proliferation capacity. Briefly, 1 × 10^4^ cells were seeded into a 96-well plate with triplicate repeats for each condition. At different time points (12, 24, 48, 72 h), 10 μl CCK-8 solution was added into each well for an additional 4 h of incubation at 37 °C. Absorbance was recorded at a wavelength of 490 nm by a microplate reader (Thermo Fisher Scientific).

### Cell Colony formation assay

For the colony formation assay, cells were seeded in six-well plates at a density of 1 × 10^2^ cells/well, followed by culturing for 15 days under standard conditions. Thereafter, cell colonies were washed with PBS twice, fixed in 70% methanol for 10 min and stained with 0.5% crystal violet for 5 min. After that, cell colonies were imaged and counted.

### Cell apoptosis assay

Flow cytometry was performed to determine the apoptosis rate by using Annexin V-FITC Apoptosis Detection Kits (BD Biosciences, USA). U2OS cells were collected after transfection and then co-incubated with Annexin V and propidium iodide for 20 min in darkness at room temperature. The samples were then analysed using a FACS analyser (Beckman Coulter, USA). The data were further analysed by using FlowJo software (Treestar, CA).

### Wound healing assay

The cells were seeded in six-well plates and incubated to reach 90% confluence in serum-free medium. A sterile pipette tip was used to create approximately 1 mm wide wounds. The detached cells were gently washed off twice, and then the medium was replaced with 1% FBS complete medium. The gap areas were imaged at 0, 24 and 48 h using a light microscope.

### Transwell assay

U2OS cells were seeded on uncoated and Matrigel-coated upper chambers for migration assays and invasion assays, respectively (BD Bioscience, USA). Culture medium that was FBS free or supplemented with 10% FBS was added to the upper and lower wells, respectively, and incubated for another 24 h. After wiping off non-migrated or non-invaded cells, the filters were fixed in 90% ethanol and stained with crystal violet. Cells in random fields were counted in each chamber using an inverted microscope (Olympus, Japan). The procedure was carried out as described previously.(Kinoshita et al. 2013)

### Total RNA extraction and qRT-PCR analyses

Total RNA was isolated by an RNA extraction kit (TaKaRa Bio, China) and reverse transcribed into cDNA by Prime Script RT Master Mix (Takara Bio). qRT-PCR was performed with a SYBR Green system (Takara) according to the following parameters: 95 °C for 10 min, followed by 40 cycles of 95 °C for 10 s and 60 °C for 50 s. The relative expression levels were calculated using the 2^−ΔΔCt^ method. For mRNA expression, GAPDH and U6 were used as internal reference controls for mRNA and miRNA expression, respectively. Primer sequences are listed in Table 1.

**Table 1:** Primer sequences for quantitative real-time PCR

Gene	Forward sequence	Reverse sequence
HULC	5′-CAGACCAAAGCATCAAGCAAGA-3’	5′-ACAAATTTGCCACAGGTTGAACA-3’
hsa-miR-372-3p	5′-CGGAAAGTGCTGCGACATTT-3’	5′-GTGCAGGGTCCGAGGT-3’
HMGB1	5′-ATATGGCAAAAGCGGACAAG-3’	5′-GCAACATCACCAATGGACAG-3’
U6	5′-CTCGCTTCGGCAGCACA-3’	5′-AACGCTTCACGAATTTGCGT-3’
GAPDH	5′-CCAGGTGGTCTCCTCTGA-3’	5′-GCTGTAGCCAAATCGTTGT-3’

### Western blot assay

Cells were harvested and lysed in RIPA lysis buffer (Beyotime, China). After determining the protein concentration using a BCA assay kit (Bio-Rad, USA), protein samples were loaded and electrophoresed in a 10% SDS-PAGE gel. Subsequently, the proteins were transferred onto nitrocellulose membranes (Millipore, USA). Membranes were blocked with 5% skimmed milk for 1 h and co-incubated with the following primary antibodies: anti-HMGB1 (1:10000, ab79823), anti-E-cadherin (1:50, ab1416), anti-N-cadherin (1:500, ab18203), anti-vimentin (1:2000, ab92547), anti-Snail (1:1500, ab53519) and anti-GAPDH (1:2500, ab9485) at 4 °C overnight. The membranes were washed three times with TBST containing 0.1% Tween 20, followed by co-incubation with HRP-labelled goat anti-mouse/rabbit IgG (1:5000, Sigma) for 2 h at room temperature. Finally, the bands were analysed by an ECL detection kit (Pierce Biotechnology, USA).

### Dual-luciferase reporter assays

The procedure was carried out as described previously.(Wang et al. 2018) Wild-type and mutant reporter plasmids of HULC (HULC-WT-luc, HULC-MUT-luc) and HMGB1 (HMGB1-WT-luc, HMGB1-MUT-luc) containing wild-type or mutant miR-372-3p mimics or mimics NC binding sites were synthesized by GenePharma (Shanghai, China). The synthesized reporter plasmids were co-transfected with miR-372-3p mimics or mimics NC by Lipofectamine 2000 (Invitrogen) when cells reached 70% confluence. The luciferase activity was analysed by the Dual-Luciferase Reporter Assay System (Promega, USA) after 48 h.

### In vivo nude mouse model for tumour formation

BALB/c nude mice were purchased from Shanghai Laboratory Animal Research Center (Shanghai, China). The nude mouse study was approved by the Animal Ethics Committee at the Third Xiangya Hospital of Central South University (Changsha, China). A total of 1 × 10^6^ U2OS cells stably transfected with sh-NC or sh-HULC were inoculated subcutaneously into nude mice. The tumour size was measured every 5 days using a Vernier scale. After 30 days, the mice were sacrificed by cervical dislocation. The tumour tissues were harvested for volume and weight measurement, qRT-PCR and western blot analysis.

### Statistical analysis

All experiments are presented as the mean ± SD, and each experiment was repeated in triplicate. Statistical analyses and graphical depictions were performed using GraphPad Prism 5.0. Student’s t test or one-way analysis of variance was performed to evaluate the group differences. Statistical significance is assessed as *p < 0.05 or **p < 0.01.

## Results

### HULC was upregulated while miR-372-3p was downregulated in osteosarcoma tissues and cell lines

**Fig. 1:**
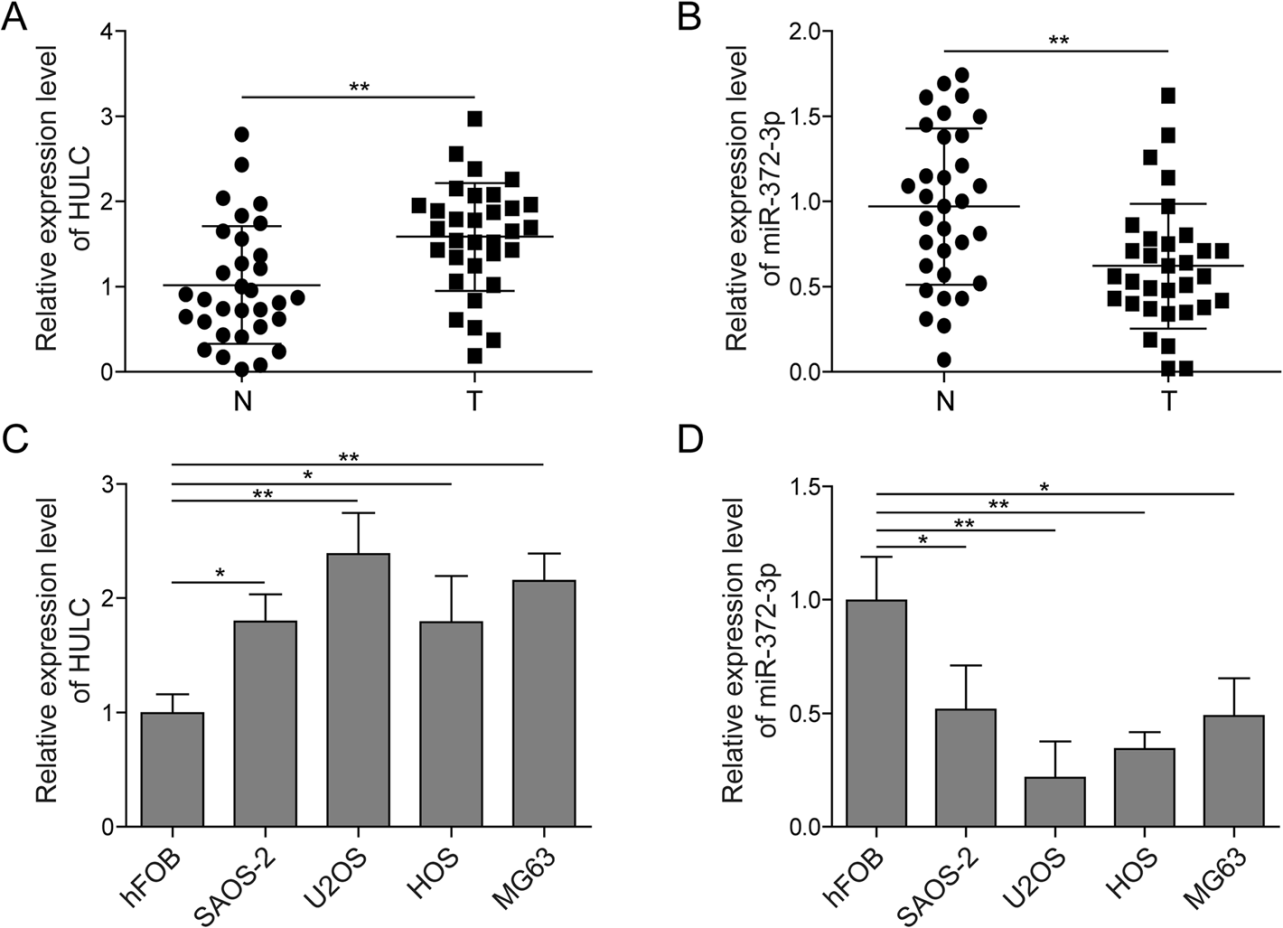
HULC was upregulated while miR-372-3p was downregulated in osteosarcoma tissues and cell lines. The expression levels of HULC and miR-372-3p were measured in osteosarcoma samples and normal tissues (*n* = 32) by qRT-PCR (A&B). The expression levels of HULC and miR-372-3p were tested in osteosarcoma cell lines (SAOS-2, U2OS, HOS, MG-63) and an osteoblastic cell line (hFOB) by qRT-PCR (C&D). Data are shown as the mean ± SD. * *p* < 0.05, ** *p* < 0.01

To investigate the regulatory role of HULC in osteosarcoma, we first asked whether HULC and miR-372-3p were dysregulated in osteosarcoma. The qRT-PCR results showed that HULC was expressed at a higher level in osteosarcoma tissues than adjacent normal tissues, while the miR-372-3p pattern was the opposite (Fig. 1a&b). In addition, we similarly assessed the HULC and miR-372-3p expression levels in four osteosarcoma cell lines (SAOS-2, U2OS, HOS, MG-63) and a normal human osteoblastic cell line (hFOB). As expected, HULC had higher expression and miR-372-3p had lower expression in osteosarcoma cell lines than hFOB cells (Fig. 1c&d). These data suggest that HULC and miR-372-3p may play important roles in regulating the development of osteosarcoma.

### HULC knockdown suppressed the proliferation, migration and invasion of osteosarcoma cells in vitro

**Fig. 2:**
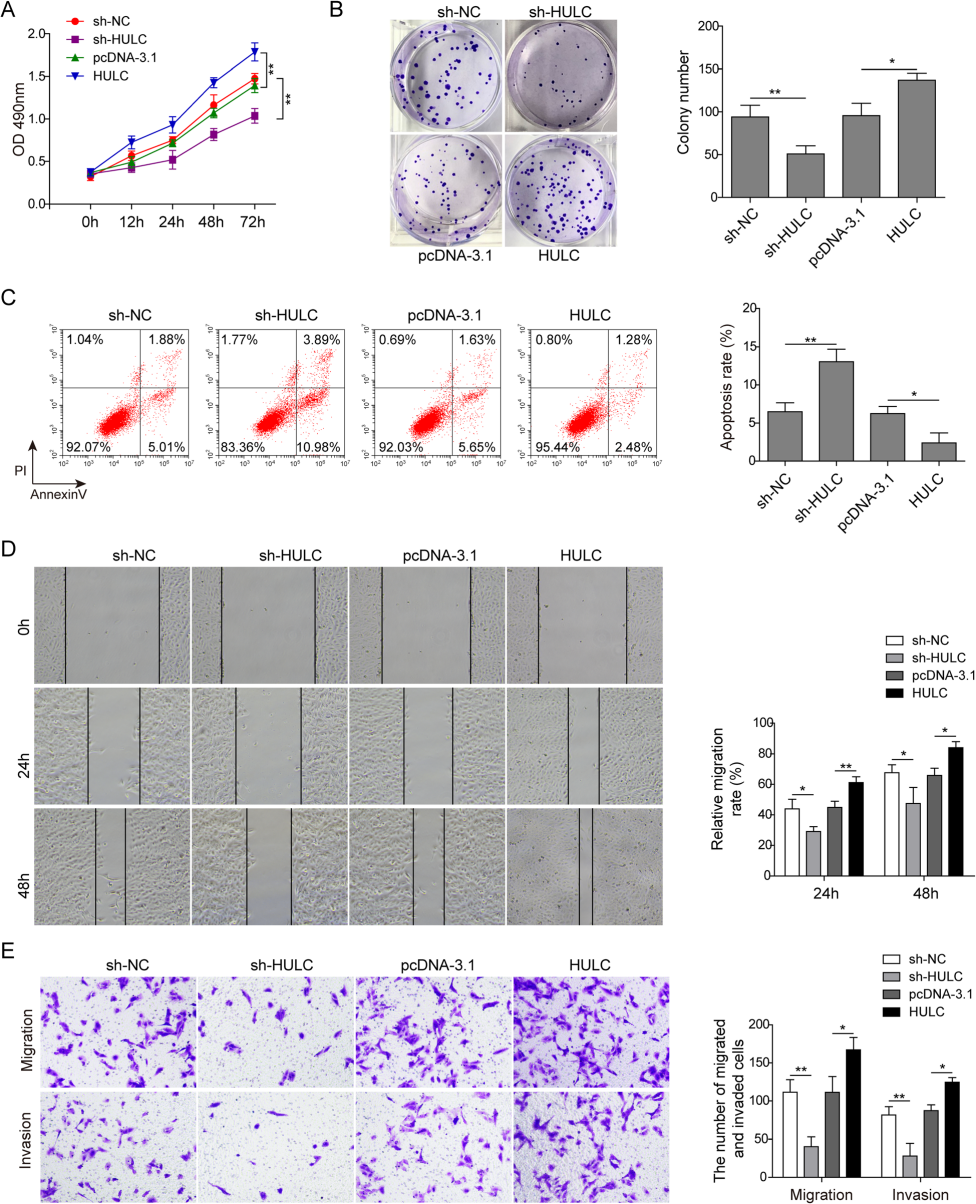
HULC knockdown inhibited the proliferation, migration and invasion of osteosarcoma cells in vitro. U2OS cells were transfected with HULC-targeting shRNA (sh-HULC), negative control shRNA (sh-NC), pcDNA3.1-HULC overexpression vector (pcDNA-HULC) or empty vector plasmid (pcDNA-3.1). Cell proliferation was tested by CCK-8 assay (A) and colony formation assay (B); cell apoptosis was analysed by flow cytometry (C); and cell migration and invasion were detected by wound healing assay (D) and transwell assay (E). Data are shown as the mean ± SD. * *p* < 0.05, ** *p* < 0.01

Next, we transfected U2OS cells with HULC shRNA (sh-HULC) or pcDNA3.1-HULC overexpression vector (HULC) to further investigate its possible impact on the behaviours of osteosarcoma cells. The negative control shRNA (sh-NC) and the empty vector plasmid (pcDNA-3.1) were used as knockdown and overexpression controls, respectively. Knockdown of HULC dramatically inhibited the proliferation of U2OS cells, which was enhanced by HULC overexpression, as evidenced by both the CCK-8 assay and colony formation assay (Fig. 2a&b). Subsequently, flow cytometry was conducted to assay cell apoptosis. The results showed that HULC knockdown clearly elevated the apoptosis rate, while overexpression of HULC strongly suppressed U2OS cell apoptosis (Fig. 2c). In addition, the migration and invasion abilities of U2OS cells were tested by wound healing and transwell assays. In the wound healing experiment, impaired migratory ability was observed after HULC depletion. In contrast, overexpression of HULC accelerated wound closure (Fig. 2d). The transwell assay also showed that HULC overexpression facilitated both the migratory and invasive abilities of U2OS cells, while HULC downregulation exerted the opposite effects (Fig. 2e). Therefore, these results indicate that HULC has oncogenic potential to induce osteosarcoma cell proliferation, migration and invasion.

### HULC functioned as a ceRNA for miR-372-3p in osteosarcoma

**Fig. 3:**
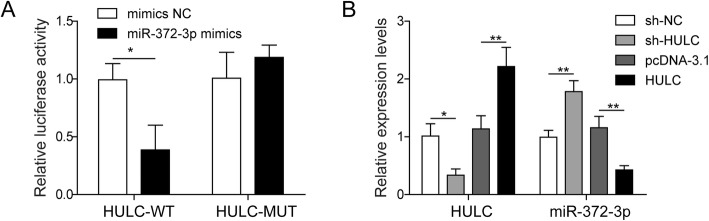
HULC served as a ceRNA for miR-372-3p in osteosarcoma. The miR-372-3p mimics and luciferase reporter plasmids with wild-type or mutant HULC 3′-UTR were co-transfected into U2OS cells. A dual luciferase reporter assay was performed to explore the direct binding relationship between HULC and miR-372-3p (A). U2OS cells were transfected with HULC-targeting shRNA (sh-HULC), negative control shRNA (sh-NC), pcDNA3.1-HULC overexpression vector (pcDNA-HULC) or empty vector plasmid (pcDNA-3.1), and then qRT-PCR was performed to evaluate the relative expression levels of HULC and miR-372-3p (B). Data are shown as the mean ± SD. * *p* < 0.05, ** *p* < 0.01

As described above, HULC and miR-372-3p were negatively correlated in osteosarcoma samples (Fig. 1a), suggesting the inhibitory effect of HULC on miR-372-3p. Dual-luciferase reporter assays were performed to verify our hypothesis. Overexpression of miR-372-3p was conducted by transfecting U2OS cells with miR-372-3p mimics. The luciferase assay results showed that miR-372-3p mimics transfection significantly decreased the luciferase signal of the reporter containing HULC-WT, but no significant effect was observed on the activity of the reporter containing HULC-MUT (Fig. 3a). Moreover, the qRT-PCR results in Fig. 3b indicated a negative regulatory relationship between HULC and miR-372-3p (Fig. 3b). Together, these results demonstrated that HULC acted as a ceRNA for miR-372-3p to inhibit miR-372-3p expression.

### miR-372-3p inhibited the proliferation, migration and invasion of osteosarcoma cells in vitro

**Fig. 4:**
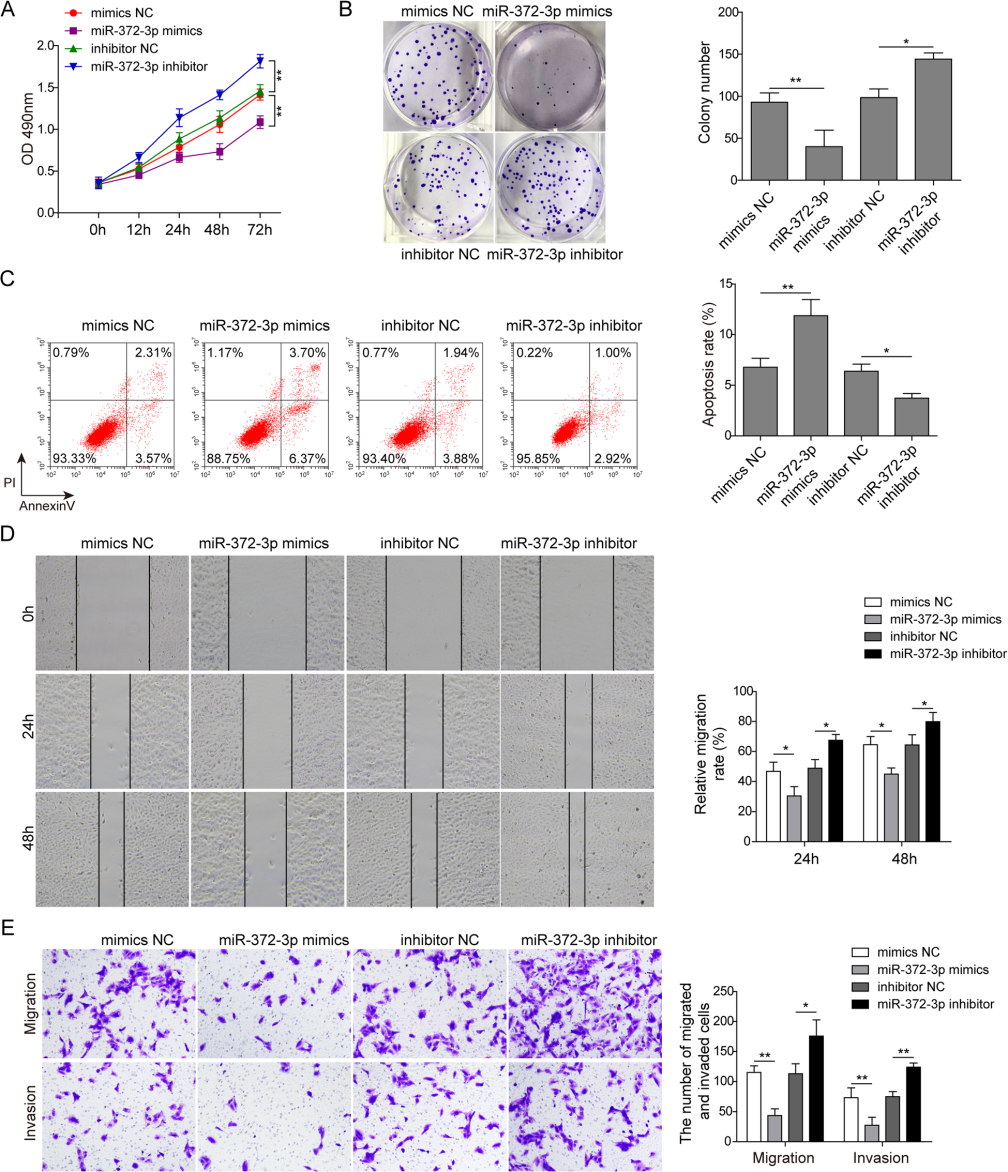
miR-372-3p suppressed the proliferation, migration and invasion of osteosarcoma cells in vitro. U2OS cells were transfected with mimics NC, miR-372-3p mimics, inhibitor NC, or miR-372-3p inhibitor. Cell proliferation was tested by CCK-8 assay (A) and colony formation assay (B); cell apoptosis was analysed by flow cytometry (C); and cell migration and invasion were detected by wound healing assay (D) and transwell assay (E). Data are shown as the mean ± SD. * p < 0.05, ** p < 0.01

To further validate the regulatory effect of miR-372-3p on the development of osteosarcoma cells, we conducted a study in which miR-372-3p mimics or inhibitor were transfected into U2OS cells. CCK-8 and colony forming assays showed an obviously decreased proliferation rate in the miR-372-3p mimics group and a significantly increased proliferation rate in the miR-372-3p inhibitor group compared with the control group (Fig. 4a&b). Flow cytometry analysis similarly indicated that miR-372-3p mimics resulted in more cell apoptosis, while apoptosis was decreased by the miR-372-3p inhibitor (Fig. 4c). The migration and invasion study further verified this trend. The migration and invasion capacity of osteosarcoma cells were inhibited upon miR-372-3p mimics transfection but increased upon inhibitor transfection (Fig. 4d&e). Collectively, these data indicated that miR-372-3p, acting as a tumour suppressor in osteosarcoma cells, could inhibit multiple malignant behaviours of U2OS cells, including cell proliferation, migration, and invasion.

### HMGB1 was a direct target of miR-372-3p

**Fig. 5:**
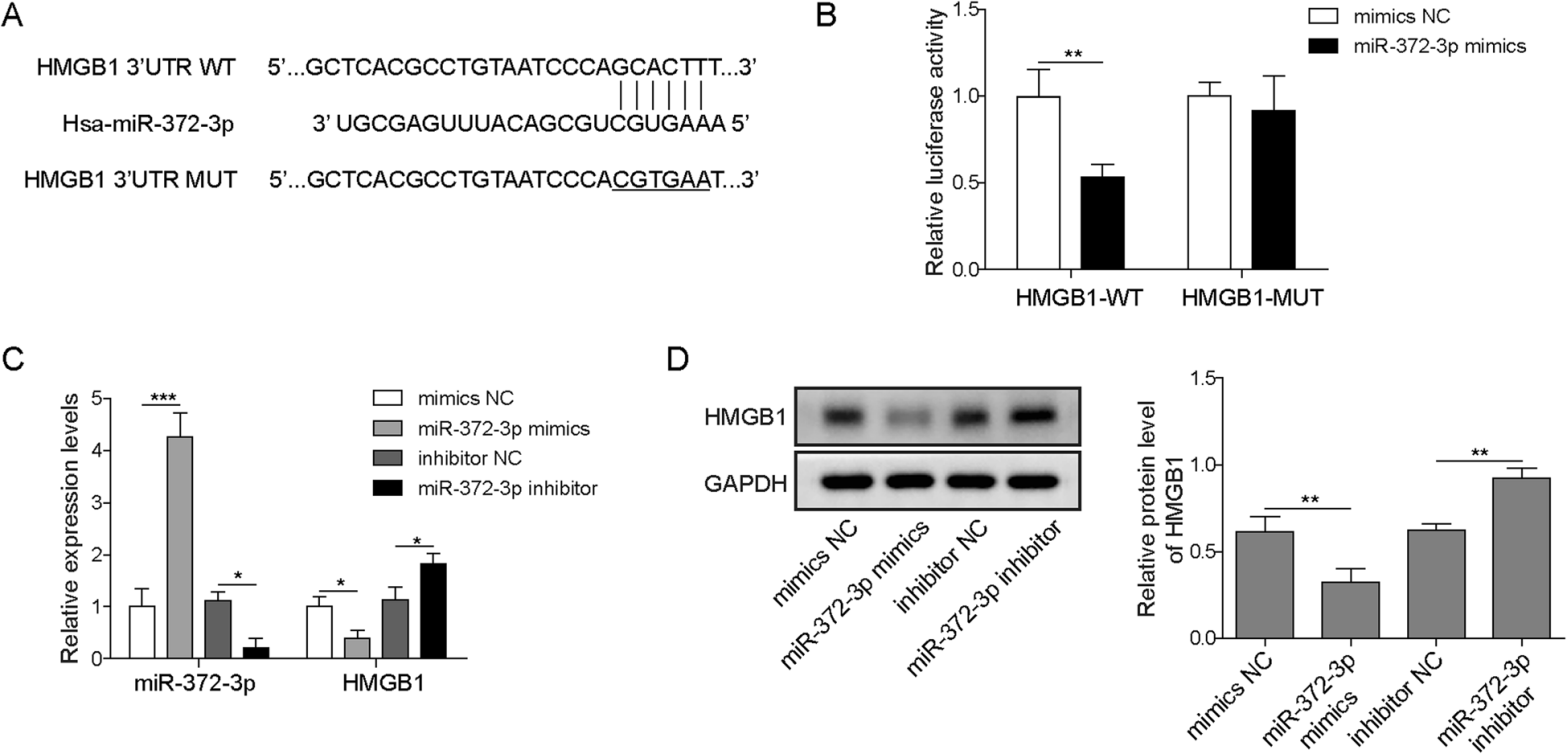
HMGB1 was a direct target of miR-372-3p. The potential binding sites between miR-373 and HMGB1 were predicted (A). The miR-372-3p mimics and luciferase reporter plasmids with wild-type or mutant HMGB1 3′-UTR were co-transfected into U2OS cells. A dual luciferase reporter assay was performed to verify the direct binding relationship between miR-372-3p and HMGB1 (B). U2OS cells were transfected with mimics NC, miR-372-3p mimics, inhibitor NC or miR-372-3p inhibitor, and then qRT-PCR was performed to evaluate the relative expression levels of miR-372-3p and HMGB1 (C). The expression level of HMGB1 was analysed by western blot (D). Data are shown as the mean ± SD. * *p* < 0.05, ** *p* < 0.01

To discover the potential target genes regulated by miR-372-3p in osteosarcoma cells, we performed bioinformatics analysis with RAID v2.0 software and identified high mobility group box 1 protein (HMGB1) as one of the target genes of miR-372 (Fig. 5a). HMGB1 has been investigated in various kinds of cancers, including osteosarcoma. However, its interaction with miR-372-3p has not yet been studied. Next, a dual-luciferase reporter assay demonstrated that the luciferase activity of cells co-transfected with miR-372-3p mimics and wild-type HMGB1 vector was notably decreased compared to that of cells co-transfected with miR-372-3p mimics and mutant HMGB1 vector (Fig. 5b). This implied that miR-372-3p probably binds to the 3′-UTR of HMGB1. Further qRT-PCR and western blot detection validated that overexpression of miR-372-3p significantly reduced HMGB1 expression, whereas knockdown of miR-372-3p significantly enhanced the expression of HMGB1 at both the mRNA and protein levels in U2OS cells (Fig. 5c&d). Taken together, these results implied that miR-372-3p was an upstream regulator to adjust HMGB1 expression.

### HULC regulated osteosarcoma cells via the miR-372-3p/HMGB1 signalling pathway

**Fig. 6:**
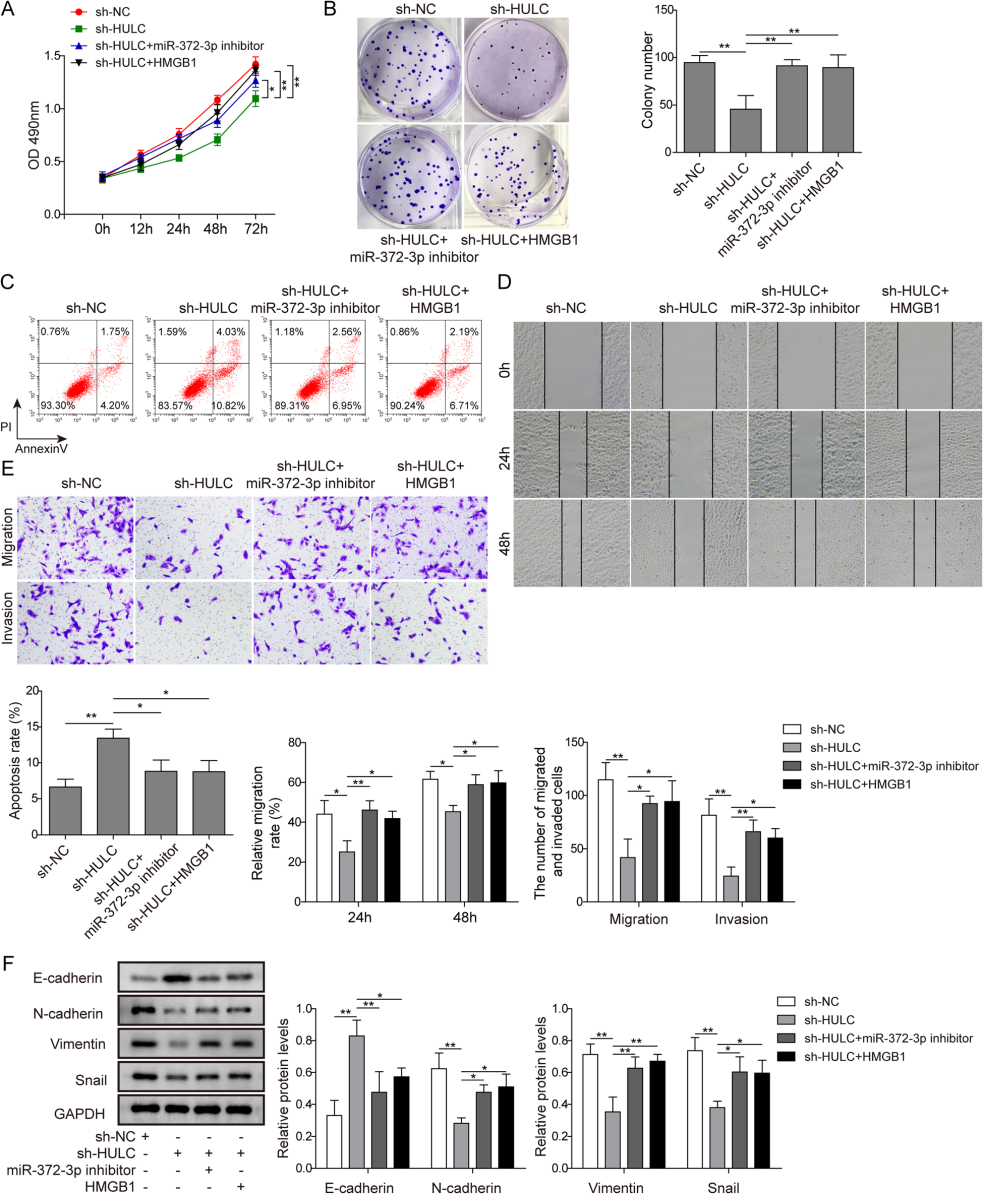
HULC regulated osteosarcoma cells via the miR-372-3p/HMGB1 signalling pathway. U2OS cells were transfected with sh-NC, sh-HULC, sh-HULC together with miR-372-3p inhibitor, or sh-HULC together with HMGB1 overexpression vector. Cell proliferation was tested by CCK-8 assay (A) and colony formation assay (B); cell apoptosis was analysed by flow cytometry (C); cell migration and invasion were detected by wound healing assay (D) and transwell assay (E); and the expression levels of E-cadherin, N-cadherin, vimentin and Snail were analysed by western blot (F). Data are shown as the mean ± SD. * *p* < 0.05, ** *p* < 0.01

We carried out rescue experiments to assess the effects of the HULC/miR-372-3p/HMGB1 pathway on U2OS cell activities. Overexpression of HMGB1 was achieved by transfecting the cells with pcDNA3.1-HMGB1. The results demonstrated that knockdown of HULC markedly reduced cell proliferation, migration and invasion and promoted cell apoptosis. However, co-transfection with sh-HULC and miR-372-3p inhibitor or HMGB1 significantly increased cell proliferation, migration and invasion and suppressed cell apoptosis compared to those in cells transfected with sh-HULC alone (Fig. 6a-e). Epithelial–mesenchymal transition (EMT) has been associated with a variety of malignant carcinoma cells; in particular, E-cadherin is considered an active suppressor of invasion and growth of many cancers.(Wheelock and Johnson 2003; Christofori 2003; Hazan et al. 2004) In the western blot study, we found that knockdown of HULC elevated E-cadherin expression levels and decreased the expression levels of N-cadherin, vimentin and Snail, but these expression levels were reversed when U2OS cells were treated with sh-HULC and miR-372-3p inhibitor or HMGB1 (Fig. 6f). Accordingly, the data suggested that the regulatory role of HULC was through the miR-372-3p/HMGB1 signalling pathway to promote the development of osteosarcoma cells.

### In vivo validation: HULC knockdown inhibited osteosarcoma tumour growth and metastasis

**Fig. 7:**
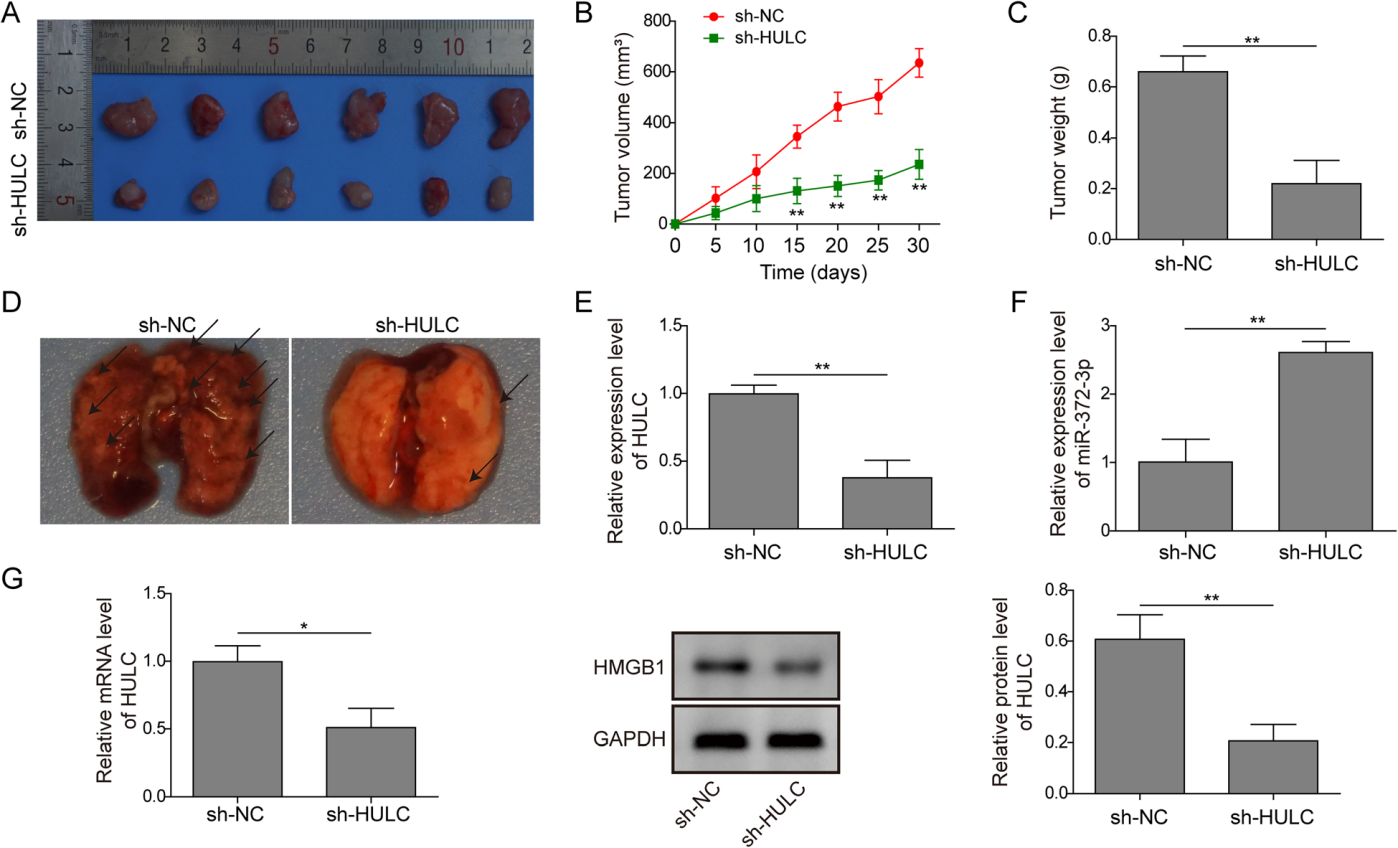
In vivo validation: HULC knockdown inhibited osteosarcoma tumour growth and metastasis. Images of tumours from the nude mouse model (A); statistical analysis of tumour volume (B) and weight (C); images of lung tissues from the nude mouse model (D); qRT-PCR or western blot was performed to evaluate the relative mRNA or protein expression levels of HULC (E), miR-372-3p (F) and HMGB1 (G). Data are shown as the mean ± SD. * p < 0.05, ** p < 0.01

Finally, we established an in vivo model to study the effect of HULC on osteosarcoma cell invasion and metastasis. As shown in Fig. 7a-c, the sh-HULC group exhibited smaller tumours of less volume or weight compared to the control group. In addition, the metastatic tumours in the lung were significantly decreased in the sh-HULC group (Fig. 7d). We further examined the tumour tissues by qRT-PCR and western blot analysis. The expression of HULC was downregulated (Fig. 7e) in the HULC-depleted tumours, accompanied by an upregulated expression of miR-372-3p (Fig. 7f) and downregulated HMGB1 (Fig. 7g). Overall, the nude mouse model confirmed that HULC could promote osteosarcoma tumour growth and metastasis by targeting miR-372-3p/HMGB1.

## Discussion

lncRNAs were initially identified in carcinogenesis due to their differential expression compared to that in normal tissues. To date, an accumulating amount of evidence has indicated that the capacity of lncRNAs to regulate complex cellular behaviours, such as cell growth and metastasis, is commonly deregulated in cancer, including osteosarcoma.(Bartonicek et al. 2016; Schmitz et al. 2016; Sallam et al. 2018) Although many potential biomarkers have been reported, specific diagnostic biomarkers for osteosarcoma have not yet been confirmed.(Bartonicek et al. 2016; Wu et al. 2014; Kong and Hansen 2009; Luetke et al. 2014; Yang et al. 2014) HULC, a novel lncRNA located on chromosome 6p24.3, has been proven to play crucial roles in the induction and progression of multiple cancers (e.g., B-cell lymphoma, human hepatocellular carcinoma, gastric cancer, colorectal cancer, and osteosarcoma) as an oncogene.(Bartonicek et al. 2016; Yu et al. 2017) In our study, we found that HULC was markedly upregulated in osteosarcoma tissues and cell lines (Fig. 1). In vitro, functional assays indicated that the depletion of HULC suppressed osteosarcoma cell proliferation, migration and invasion but induced apoptosis (Fig. 2), demonstrating the potential of HULC as a therapeutic target for osteosarcoma intervention. Therefore, we next explored the underlying mechanism in osteosarcoma cell lines.

The ceRNA theory was first proposed in 2011 and subsequently extensively accepted in the noncoding RNA field.(Salmena et al. 2011) By this mechanism, lncRNAs may serve as ceRNAs by sponging miRNAs to inhibit their targeting effect. Bioinformatics analyses revealed that there was a conserved binding site of miR-372-3p on HULC. Therefore, we hypothesized that HULC could affect miR-372-3p via a ceRNA mechanism. In an encouraging result, we validated that miR-372-3p did have a reciprocal suppressive effect on HULC expression (Fig. 1) and that knockdown of miR-372-3p induced the proliferation, migration and invasion of osteosarcoma cells in vitro (Fig. 4). Importantly, the dual-luciferase assay further showed that HULC directly interacts with miR-372-3p to reduce its expression (Fig. 3), suggesting that HULC served as a miRNA sponge that could bind to and regulate miR-372-3p expression.

miRNAs control gene expression by binding to the 3′-UTR of the target gene, which causes mRNA cleavage or translational repression.(Catalanotto et al. 2016; Valinezhad Orang et al. 2014) To explore the target genes of miR-372-3p, RAID v2.0 software was used and identified HMGB1 as one of the targets of miR-372-3p. HMGB1, which is overexpressed in CRC, pancreatic cancer, hepatoma and leukaemia, has been reported to strongly affect tumour progression, lymph node size and metastatic infiltration.(Kang et al. 2014) Previous reports clarified that the significantly increased HMGB1 expression in osteosarcoma cells promoted autophagy, which led to apoptosis inhibition and increased drug resistance.(Huang et al. 2012a; Huang et al. 2012b) Typically, the suppression of HMGB1 increases sensitivity to chemotherapy in vitro, implicating HMGB1 as a potential ideal target to improve osteosarcoma therapy.(Huang et al. 2012b) In our study, dual-luciferase assays confirmed the direct interaction between miR-372-3p and HMGB1 (Fig. 5). Moreover, HMGB1 was negatively regulated by miR-372-3p. Further rescue experiments showed that HULC promoted proliferation and metastasis in osteosarcoma via regulation of the miR-372-3p/HMGB1 signalling axis (Fig. 6). In addition, the in vivo nude mouse study consistently demonstrated the same tumour growth trend as the in vitro findings when HULC was depleted (Fig. 7).

miR-372-3p was recently reported to be regulated by lncRNA OSER1-AS1 to positively affect Rab23 expression and ultimately suppress tumour progression in hepatocellular carcinoma (HCC).(Fan et al. 2019) In our study, we mainly explored the functional roles and mechanism of the HULC/miR-372-3p/HMGB1 pathway in osteosarcoma, which has never been identified before. In fact, miRNAs could exhibit powerful effects on the development of malignancy due to their potential to control numerous target genes.(Catalanotto et al. 2016) However, this phenomenon is also a limitation in miRNA research, and we may continue to explore other pathways in subsequent research.

Although we initially revealed a novel downstream regulatory mechanism of osteosarcoma cells involving HULC both in vitro and in vivo, to deeply understand this complex mechanism, much remains to be implemented. Future studies are needed to further explore whether there are other pathways involved in osteosarcoma progression.

## Conclusions

The present study is the first to demonstrate that HULC acts as a novel oncogene in osteosarcoma both in vitro and in vivo. Furthermore, HULC acts as a ceRNA to regulate HMGB1 expression by competitively sponging miR-372-3p, thereby regulating the progression of osteosarcoma. Therefore, our findings provide useful information to identify new biomarkers for early diagnosis and therapeutic application in osteosarcoma progression.

## Data Availability

All data generated or analysed during this study are included in this published article.

## Abbreviations

CRC: Colorectal cancer
ceRNAs: Competing endogenous RNAs
EMT: Epithelial–mesenchymal transition
HMGB1: High mobility group box 1 protein
HULC: Highly upregulated in liver cancer
HOTAIR: Hox transcript antisense intergenic RNA
lncRNAs: Long non-coding RNAs
miRNAs: MicroRNAs
qRT-PCR: Quantitative real-time polymerase chain reaction
shRNA: Short hairpin RNA
3’UTR: 3′ untranslated region
HCC: hepatocellular carcinoma

## References

[CR1] Ardekani AM, Naeini MM (2010). The role of MicroRNAs in human diseases. Avicenna J Med Biotechnol.

[CR2] Bartel B (2005). MicroRNAs directing siRNA biogenesis. Nat Struct Mol Biol.

[CR3] Bartel DP (2004). MicroRNAs: genomics, biogenesis, mechanism, and function. Cell.

[CR4] Bartonicek N, Maag JLV, Dinger ME (2016). Long noncoding RNAs in cancer: mechanisms of action and technological advancements. Mol Cancer.

[CR5] Bielack SS, Kempf-Bielack B, Delling G (2002). Prognostic factors in high-grade osteosarcoma of the extremities or trunk: an analysis of 1,702 patients treated on neoadjuvant cooperative osteosarcoma study group protocols. J Clin Oncol.

[CR6] Catalanotto C, Cogoni C, Zardo G (2016). MicroRNA in control of gene expression: an overview of nuclear functions. Int J Mol Sci.

[CR7] Christofori G (2003). Changing neighbours, changing behaviour: cell adhesion molecule-mediated signalling during tumour progression. EMBO J.

[CR8] Croce CM, Calin GA (2005). miRNAs, cancer, and stem cell division. Cell.

[CR9] Fan J, Zhang J, Huang S, Li P. lncRNA OSER1-AS1 acts as a ceRNA to promote tumorigenesis in hepatocellular carcinoma by regulating miR-372-3p/Rab23 axis. *Biochemical and biophysical research communications.* 2019. 10.1016/j.bbrc.2019.10.105 31635804

[CR10] Harrison DJ, Geller DS, Gill JD, Lewis VO, Gorlick R (2018). Current and future therapeutic approaches for osteosarcoma. Expert Rev Anticancer Ther.

[CR11] Hazan RB, Qiao R, Keren R, Badano I, Suyama K (2004). Cadherin switch in tumor progression. Ann N Y Acad Sci.

[CR12] Huang J, Liu K, Yu Y (2012). Targeting HMGB1-mediated autophagy as a novel therapeutic strategy for osteosarcoma. Autophagy.

[CR13] Huang J, Ni J, Liu K (2012). HMGB1 promotes drug resistance in osteosarcoma. Cancer Res.

[CR14] Isakoff MS, Bielack SS, Meltzer P, Gorlick R. Osteosarcoma: Current Treatment and a Collaborative Pathway to Success 2015;33(27):3029–3035. 10.1200/JCO.2014.59.4895 PMC4979196 26304877

[CR15] Kang R, Chen R, Zhang Q (2014). HMGB1 in health and disease. Mol Asp Med.

[CR16] Kinoshita T, Nohata N, Hanazawa T (2013). Tumour-suppressive microRNA-29s inhibit cancer cell migration and invasion by targeting laminin-integrin signalling in head and neck squamous cell carcinoma. Br J Cancer.

[CR17] Kong C, Hansen MF (2009). Biomarkers in osteosarcoma. Expert Opin Med Diagn.

[CR18] Kong D, Wang Y (2018). Knockdown of lncRNA HULC inhibits proliferation, migration, invasion, and promotes apoptosis by sponging miR-122 in osteosarcoma. J Cell Biochem.

[CR19] Lee HE, Park KU, Yoo SB (2013). Clinical significance of intratumoral HER2 heterogeneity in gastric cancer. Eur J Cancer.

[CR20] Li Z, Dou P, Liu T, He S (2017). Application of long noncoding RNAs in osteosarcoma: biomarkers and therapeutic targets. Cell Physiol Biochem.

[CR21] Liang W-C, Fu W-M, Wong C-W (2015). The lncRNA H19 promotes epithelial to mesenchymal transition by functioning as miRNA sponges in colorectal cancer. Oncotarget.

[CR22] Liu X-h, Sun M, Nie F-Q, et al. Lnc RNA HOTAIR functions as a competing endogenous RNA to regulate HER2 expression by sponging miR-331-3p in gastric cancer. 2014;13(1):92. 10.1186/1476-4598-13-92 PMC4021402 24775712

[CR23] Luetke A, Meyers PA, Lewis I, Juergens H (2014). Osteosarcoma treatment – where do we stand? A state of the art review. Cancer Treat Rev.

[CR24] Sallam T, Sandhu J, Tontonoz P (2018). Long noncoding RNA discovery in cardiovascular disease: decoding form to function. Circ Res.

[CR25] Salmena L, Poliseno L, Tay Y, Kats L, Pandolfi PP (2011). A ceRNA hypothesis: the Rosetta stone of a hidden RNA language?. Cell.

[CR26] Schmitz SU, Grote P, Herrmann BG (2016). Mechanisms of long noncoding RNA function in development and disease. Cell Mol Life Sci.

[CR27] Valinezhad Orang A, Safaralizadeh R, Kazemzadeh-Bavili M. Mechanisms of miRNA-Mediated Gene Regulation from Common Downregulation to mRNA-Specific Upregulation %J International Journal of Genomics. 2014;2014:15. 10.1155/2014/970607 PMC4142390 25180174

[CR28] Wang J, Liu X, Wu H (2010). CREB up-regulates long non-coding RNA, HULC expression through interaction with microRNA-372 in liver cancer. Nucleic Acids Res.

[CR29] Wang Q, Liu S, Zhao X, Wang Y, Tian D, Jiang W (2017). MiR-372-3p promotes cell growth and metastasis by targeting FGF9 in lung squamous cell carcinoma. Cancer Med.

[CR30] Wang Y, Huang Y, Xiang P, Tian W (2017). LncRNA expression and implication in osteosarcoma: a systematic review and meta-analysis. Oncotargets Ther.

[CR31] Wang Y, Lu Z, Wang N (2018). Long noncoding RNA DANCR promotes colorectal cancer proliferation and metastasis via miR-577 sponging. Exp Mol Med.

[CR32] Wheelock MJ, Johnson KR (2003). Cadherins as modulators of cellular phenotype. Annu Rev Cell Dev Biol.

[CR33] WU DAJIANG, CHEN KAI, BAI YUSHU, ZHU XIAODONG, CHEN ZIQIANG, WANG CHUANFENG, ZHAO YINGCHUAN, LI MING (2014). Screening of diagnostic markers for osteosarcoma. Molecular Medicine Reports.

[CR34] Xu SY, Xu PF, Gao TT (2018). MiR-372-3p inhibits the growth and metastasis of osteosarcoma cells by targeting FXYD6. Eur Rev Med Pharmacol Sci.

[CR35] Yang Guodong, Lu Xiaozhao, Yuan Lijun (2014). LncRNA: A link between RNA and cancer. Biochimica et Biophysica Acta (BBA) - Gene Regulatory Mechanisms.

[CR36] Yu X, Zheng H, Chan MTV, WKK W. HULC: an oncogenic long non-coding RNA in human cancer. 2017;21(2):410–7. 10.1111/jcmm.12956 PMC5264137 27781386

